# Serine 39 in the GTP‐binding domain of Drp1 is involved in shaping mitochondrial morphology

**DOI:** 10.1002/2211-5463.13820

**Published:** 2024-05-17

**Authors:** Marvi Ghani, Bernadett Szabó, Mahmoud Alkhatibe, Hailemariam Amsalu, Peleg Zohar, Eszter Anna Janka, János András Mótyán, Krisztina Tar

**Affiliations:** ^1^ Department of Medical Chemistry, Faculty of Medicine University of Debrecen Hungary; ^2^ Doctoral School of Molecular Medicine University of Debrecen Hungary; ^3^ Department of Dermatology, MTA Centre of Excellence, Faculty of Medicine University of Debrecen Hungary; ^4^ HUN‐REN‐UD Allergology Research Group University of Debrecen Hungary; ^5^ Department of Biochemistry and Molecular Biology, Faculty of Medicine University of Debrecen Hungary

**Keywords:** Drp1, mitochondrial network collapse, S39A, single amino acid mutagenesis

## Abstract

Continuous fusion and fission are critical for mitochondrial health. In this study, we further characterize the role played by dynamin‐related protein 1 (Drp1) in mitochondrial fission. We show that a single amino acid change in Drp1 at position 39 from serine to alanine (S39A) within the GTP‐binding (GTPase) domain results in a fused mitochondrial network in human SH‐SY5Y neuroblastoma cells. Interestingly, the phosphorylation of Ser‐616 and Ser‐637 of Drp1 remains unaffected by the S39A mutation, and mitochondrial bioenergetic profile and cell viability in the S39A mutant were comparable to those observed in the control. This leads us to propose that the serine 39 residue of Drp1 plays a crucial role in mitochondrial distribution through its involvement in the GTPase activity. Furthermore, this amino acid mutation leads to structural anomalies in the mitochondrial network. Taken together, our results contribute to a better understanding of the function of the Drp1 protein.

## Graphical abstract

Drp1 is one of the critical proteins of mitochondrial fission. We studied the effects of the loss‐of‐function mutation of serine 39 in the GTPase domain of Drp1 in an *in vitro* cellular model. The mutation results in elongated mitochondria, leading to structural anomalies in the mitochondrial network. We propose that serine 39 of Drp1 is crucial in mitochondrial distribution.

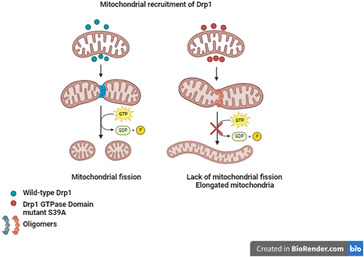

**Abbreviations**
AD: Alzheimer's disease
CaMKII: calmodulin‐dependent protein kinase II
Cdk1: cyclin‐dependent kinase 1
DMEM: Dulbecco's Modified Eagle Medium
Drp1: dynamin‐related protein 1
EDTA: ethylenediaminetetraacetic acid
FCCP: carbonyl cyanide 4‐(trifluoromethoxy)phenyl hydrazone
Fis1: mitochondrial fission protein 1
GED: GTPase effector domain
GSK3β: glycogen synthase kinase
GTP: guanosine triphosphate
HEK293T: human embryonic kidney 293T
MD: middle domain
MFF: mitochondrial fission factor
MFN1: mitofusin 1
MFN2: mitofusin 2
MiD49: mitochondrial dynamic protein of 49
MiD51: mitochondrial dynamic protein of 51
mtDNA: mitochondrial DNA
OCR: oxygen consumption rate
Opa1: optic atrophy factor 1
PAGE: polyacrylamide gel electrophoresis
PBS: phosphate‐buffered saline
PFA: paraformaldehyde
PKA: cAMP‐dependent protein kinase
PKC: protein kinase *C*
PMSF: phenylmethylsulfonyl fluoride
PTM: post‐translational modification
ROCK: Rho‐associated protein kinase
SDS: sodium dodecyl suplhate
Ser: serine
TEM: transmission electron microscopy
WT: wild‐type

Fusion and fission are continuous processes to maintain the healthy state of the mitochondria. Unopposed fusion or fission reduces mitochondrial functionality in response to deleting specific factors [1, 2]. The fusion of mitochondria promotes repair and complementation processes, while damaged mitochondria are segregated from the network by fission [3, 4]. A family of proteins called Dynamin superfamily proteins ensures the proper functioning of mitochondria by regulating fusion and fission processes. The dynamin‐related protein 1 (Drp1) is a cytosolic protein that plays a pivotal role in mitochondrial and peroxisome fission, synapse formation, and cell death [5, 6]. Mitochondrial fission, predominately regulated by the GTPase activity of Drp1, provides quality control for the organelle [7, 8].

Drp1 and its yeast ortholog Dnm1 are large dynamin‐related mechanochemical enzymes that use their GTPase activity to drive membrane fission [9, 10, 11]. A part of the cytosolic Drp1 pool is recruited by adaptor proteins to be bound to the mitochondria to initiate fission [12]. Several of these proteins have been identified, including the mitochondrial fission protein (Fis1), mitochondrial dynamic proteins of 49 and 51 kDa (MiD 49, MiD51), and the mitochondrial fission factor (MFF) [13]. Unopposed fusion, on the other hand, is mainly regulated by mitofusins (MFN1 and MFN2) and the optic atrophy factor 1 (Opa1) [14, 15, 16, 17, 18, 19].

Dnm1/Drp1 proteins share the classical dynamin property to assemble into oligomeric structures [20, 21]. The structure of Drp1 is composed of a conserved N‐terminal GTP‐binding domain (GTPase domain), a middle domain (MD) involved in self‐assembly into an oligomeric structure, a variable PH‐domain, and at the C terminus a GTPase effector domain (GED), which takes part in both inter‐ and intramolecular interactions. Dynamins have a high GTPase activity and low GTP affinity, which, along with regulatory proteins, allow careful modulation between the activated GTP‐bound and the inactivated GDP‐bound state. The GTPase domain of Drp1 was systematically mapped, and multiple residues have been characterized as essential elements for the GTPase activity, including Ser39 in the phosphate‐binding loop, where the α‐phosphate is bound via primary and side‐chain interaction by Ser40 and by backbone interactions of Ser39 and Lys38. The hydroxyl group of Ser39 was found to rotate about 180° upon nucleotide occupation of the active site. Ser39 does not make significant interactions with the β‐phosphate binding [22]. Single amino acid mutagenesis of Ser39 within the GTPase domain completely abolished the GTPase activity of Drp1, similar to the well‐studied K38A mutation [22, 23, 24, 25].

The number of the characterized S39 variants is limited; only the S39A, S39I, and S39N variants have been investigated. The S39A mutation is known to abolish GTP hydrolysis [22] completely. The S39N mutant protein—having reduced affinity for GTP—caused reduced peroxisome abundance in the cells expressing this mutant [26]. In contrast, the S39I mutant caused altered localization, at least in the perinuclear region (information about its GTPase activity is unavailable) [27]. In addition, the mutation of the consensus sequence motif in the corresponding position of dynamin‐1 (S45N mutation) was also reported to cause a loss of GTPase activity [28].

The S39 residue constitutes a part of a consensus motif in the P‐loop/G1. Because this sequence motif of the dynamin superfamily is highly conserved, mutations in this position might have a negative effect on GTPase activity, and gain‐of‐function mutations have not been reported for this position so far. Accordingly, the mutation of dynamin‐1's S45 residue was expected to disrupt important contacts required for nucleotide binding [29]; therefore, mutations of the S39 residue might not be expected to improve the catalytic activity.

Reversible phosphorylation is one of the post‐translational modifications of Drp1. The two phosphorylation sites that have been extensively studied are located at Ser‐616 and Ser‐637 of the protein. Phosphorylation of Ser‐616 is likely to activate fission, and phosphorylation of Ser‐637 is mainly an inactivating step [30]. Cdk1‐catalyzed phosphorylation of Drp1 at Ser‐616 promotes its translocation to the mitochondria, leading to mitochondrial division in Jurkat 6E.1 cells [31]. Other protein kinases, such as Rho‐associated protein kinase (ROCK), protein kinase *C* (PKC), ERK1/2, and calmodulin‐dependent protein kinase II (CaMKII), also phosphorylate Ser‐616 in a variety of cells [32, 33, 34]. Drp1 is phosphorylated at Ser‐637 by a variety of kinases, including PKA. The specific role of phosphorylation depends on several parameters, including cell type and upstream regulatory molecules. In most cases, however, phosphorylation of Ser‐637 of Drp1 leads to a reduction in the catalytic activity of the protein [35, 36].

Mitochondrial and cellular homeostasis depends on both mitochondrial fusion and fission. Any disturbance in the homeostatic equilibrium caused by cellular stimuli may result in elongated or fragmented mitochondria, which are associated with pathological disorders. Thus, mitochondrial homeostasis demonstrates the physiological significance of mitochondrial fusion and fission [3].

Variable mitochondrial morphologies specific to different tissues and cells and their particular functionality have been identified. For example, in liver cells, the mitochondrial structures are described to be compact spherical shaped, which, upon cellular stress, change their formation to c‐shaped [37]. In mice, the mitochondria exhibit tubular elongated structure in the brain's white matter; with aging, the number of mitochondria decreases, accompanied by an increase in length [38]. Emerging studies have shown the development of machine learning‐based methods and have proven the necessity of the applications of mitochondrial classification in different tissues, including brain tissue and cells such as primary neurons, differentiating neurons, and neuroblastoma cells [39, 40, 41].

Several findings have indicated that mitochondrial dynamics are essential in mitochondrial life. An exchange of components of the different mitochondria can be achieved through their encounters, and the damaged parts and enzymes can be set aside through fission processes in a selective manner. Therefore, a functional pool of mitochondria can be maintained by this reorganization. Through multiple studies, mitochondrial dynamics have become apparent to have a crucial role in quality control and the adaptation to the bioenergetics needs of the cell [42].

Post‐translational modifications, altered levels of gene expression, loss of regulation, or direct mutations affect the structure and function of Drp1, hence mitochondrial function contributing to the onset of pathogenesis. Drp1 is highly recognized for its involvement with neurodegenerative diseases [6, 19, 43, 44, 45, 46]. Neurodegenerative diseases are often associated with mitochondrial dysfunction. Aggregation‐prone proteins such as the huntingtin protein in Huntington's disease, α‐synuclein in Parkinson's disease, or mutant AD proteins in Alzheimer's disease abnormally interact with Drp1, leading to increased activity of Drp1, access of mitochondrial fragmentation and damage in neurons. Currently, a significant focus is to prevent this abnormal interaction by developing and testing drugs that target to inhibit or reduce these interactions [47], characterize cells upon applying drugs that reduce mitochondrial fragmentation by reducing the activity of Drp1 [48], genetic manipulation of Drp1 level [40] and describe mitochondrial abnormalities in diseased models [49, 50, 51].

Pathogenic mutations of Drp1 were described in the GTPase and stalk domains. These *de novo* mutations represent a wild clinical spectrum of disorders [19]. The first reported pathogenic mutation of Drp1 was a point mutation in alanine 395 to aspartic acid. The mutation expressed association with the death of newborns due to multiple developmental disorders such as optic atrophy, microencephaly, and production of very long‐chain fatty acids [52]. *De novo* variants affecting the GTPase domain have only been reported in five clinical cases [23, 53, 54, 55, 56]. These disease‐associated mutations throughout the GTPase and middle domains of Drp1 were analyzed by generating and using recombinant proteins containing the specific mutation. The functional study demonstrated that the functional defects caused by mutations in Drp1 are highly variable [57]. Dominant‐negative mutants in the GTP binding domain affect the enzyme's catalytic activity, leading to a complete loss of function. Recently, Keller *et al*. [56] presented a case study where they reported that a heterozygous missense variant of Drp1 (Ser39 to Gly in the GTPase domain) resulted in a severe neurodevelopmental disorder presenting with severe amyotrophy, dystonia, and sensory neuropathy in a 10‐year‐old male. However, follow‐up mitochondrial morphology studies still need to be done to investigate the effect of mutation on mitochondrial rearrangement.

In this study, we demonstrate that a mutation of Drp1 at Ser39 within the GTP‐binding domain's phosphate‐binding loop (G1) generates an elongated, bulky mitochondrial network. The S39A mutation did not affect the phosphorylation of the distant Ser‐616 and Ser‐637 amino acid residues of Drp1. Compared with cells overexpressing the wild‐type Drp1, the Drp1 S39A mutant shows mitochondrial bioenergetics and cell viability similar to control. We conclude that the Ser39 amino acid residue is involved in the mitochondrial morphology arrangement of human SH‐SY5Y neuroblastoma cells *in vitro*.

## Materials and methods

All materials were purchased from Sigma‐Aldrich (St. Louis, MO, USA) unless otherwise specified.

### Cell culture

Modified human SH‐SY5Y (European Tissue Culture/Sigma cat# 94030304) cells were maintained in DMEM with high glucose, supplemented with 10% fetal bovine serum (FBS), 2 mm l‐glutamine, and 1× (vol/vol) antibiotic‐antimycotic (Gibco, Thermo Fischer Scientific, Waltham, MA, USA), at 37 °C in a 5% CO_2_ incubator. HEK293T cells were cultured in DMEM supplemented with 10% FBS, 2 mm l‐glutamine, and 1× (vol/vol) antibiotic‐antimycotic (Gibco) at 37 °C in a 5% CO_2_ incubator.

### Quick change site‐directed mutagenesis

According to the manufacturer's instructions, the mCherry‐Drp1 S39A mutant was prepared using Quick Change site‐directed mutagenesis (cat. 200523, Agilent Technologies, Santa Clara, CA, USA). We used the mCherry‐Drp1 plasmid (the mCh‐Drp1 was a gift from Gia Voeltz (Addgene plasmid #49152; http://n2t.net/addgene:49152; RRID: Addgene_49152)) [58] as a template with the following primers: 5′ GCTTTCTAGCACTGAGGCCTTTCCGCTGCTCTGC 3′ and 5′ GCAGAGCAGCGGAAAGGCCTCAGTGCTAGAAAGC 3′. The mutation was verified by sequencing (Center for Clinical Genomics and Personalized Medicine, Core Facility, University of Debrecen). The mCherry control vector was a gift from Dr György Vámosi (Department of Biophysics and Cell Biology, Faculty of Medicine, University of Debrecen, Hungary).

### Transfection and generation of stable cell lines

Cells with the mCherry control, mCherry‐Drp1, and mCherry‐Drp1 S39A plasmids were transfected using Lipofectamine 3000 (cat. L3000001, Thermo Fischer Scientific) according to the manufacturer's protocol. Transfection efficiency was monitored by detecting the expression of mCherry. After 72 h of transfection, cells were subjected to 800 μg·mL^−1^ geneticin antibiotic selection (cat. 11811023, ThermoFischer Scientific) to generate cell lines stably expressing the recombinant proteins. The titration of antibiotics and the scale‐up of cells were performed as we previously described [59].

### Western blotting

Cells were washed with 1× phosphate‐buffered saline (PBS) and lysed in RIPA buffer (50 mm Tris–HCl pH 7.4, 150 mm NaCl, 0.5% Na‐deoxycholate, 2 mm EDTA, 1% NP‐40, and 50 mm NaF) supplemented with a protease inhibitor cocktail (1 mm benzamidine, 1 mm PMSF, and cOmplete Mini‐EDTA‐free protease inhibitor cocktail (Merck, Darmstadt, Germany)). Cells were centrifuged at 13 400 *g* at 4 °C for 15 min. The supernatants were collected, and the protein concentration was estimated using a Bradford protein assay. Proteins (20 μg per well) were resolved on standard SDS/polyacrylamide gel electrophoresis (PAGE) and transferred to a nitrocellulose membrane. Membranes were incubated with primary antibodies (anti‐DLP1 primary antibody, BD Biosciences (Becton Drive Franklin Lakes, NJ, USA), cat. 611113, dilution 1 : 1000, phospho‐Drp1 Ser 616 antibody Cell Signaling (Danvers, MA, USA) cat. 3455, dilution 1 : 500, phospho‐Drp1 Ser 637 antibody Cell Signaling cat. 6319, dilution 1 : 500) overnight at 4 °C and subsequently incubated with an HRP‐conjugated secondary antibody for 2 h at room temperature (RT). β‐Actin was used for internal loading control β‐actin (C4), Santa Cruz Biotechnology cat no. SC‐47778, dilution 1 : 3000 or β‐actin (C4) HRP, cat. SC‐47778 HRP, dilution 1 : 2000. Blots were developed with enhanced chemiluminescence (Santa Cruz Biotechnology, Dallas, TX, USA) and captured by ChemiDoc Imager (Bio‐Rad Laboratories, Hercules, CA, USA).

### Nuclei staining of fixed cells

Twelve‐well plates were coated with 1% gelatin before cell seeding. Cells were seeded to reach 70% confluency the next day. Following transient transfection, cells were incubated for 72 h. The cells were then rinsed with 1× PBS three times, fixed with 3.7% paraformaldehyde (PFA) for 15 min, and permeabilized with 0.1% Triton X‐100 in PBS for 30 min. The nuclei were counterstained with 1 μg·mL^−1^ DAPI. Coverslips were mounted on slides with mounting media (Dabco 33‐LV: Mowiol 4‐88, 1 : 50). Images were taken with an SP8 confocal laser scanning microscope (Leica Biosystems, Wetzlar, Germany) using a 63× HC PL Apo oil CS2 objective.

### Mitochondrial staining for live cell high content analysis confocal imaging

Cells stably expressing the mCherry, mCherry‐Drp1 WT, and mCherry‐Drp1 S39A were seeded at the density of 1.5 × 10^4^ cell per well in a cell carrier 96 ultra microplates (PerkinElmer, Waltham, MA, USA) in DMEM with high glucose. The following day, cells were carefully rinsed with 1× PBS and incubated with 50 nm freshly diluted Mitotracker Green (ThermoFischer Scientific) and 10 μm Hoechst 33342 in serum‐free media for 20 min at 37 °C in a 5% CO_2_ incubator. The media was changed to fresh culture media, and live cell imaging was performed using an Opera Phoenix High Content Screening system (PerkinElmer) at 37 °C at 5% CO_2_.

### High content analysis

Image‐acquisition settings were 63× water objective (NA = 1.15), appropriate lasers and filters for Hoechst, Mitotraker Green, and mCherry in sequential mode to exclude spectra overlap. Detection was performed with a 16‐bit camera under nonsaturating conditions.

After selecting the optimal z‐frame position, the detection was done with a 16‐bit camera with non‐saturating conditions. Images were analyzed using the harmony 4.9 software (PerkinElmer), and different fluorescence staining was used for cell segmentation (both the mCherry and MitoTracker Green were used to identify the cytoplasm). Hoechst determines the nuclei, Mitotracker Green determines the mitochondrial compartments, and cytoplasm defines the premises of each cell. Analysis was performed with the harmony 4.9 software. The true nuclei were defined based on the Hoechst channel. The mitochondria were classified through identification from deconvolved and preprocessed images using the find spots algorithm in PhenoLOGIC machine learning. As mitochondria display a variety of shapes, five categories were established: hyperfuse, round, compact tubular, long tubular, short tubular, and fragmented. Mitochondria morphology analysis was performed as previously described [40, 41, 60].

### Transmission electron microscopy (TEM)

Monolayers of mCherry control, mCherry‐Drp1 WT, and mCherry‐Drp1 S39A cells were grown on Aclar thermoplastic film (EMS‐Electron Microscopy Sciences). Cell pellets were fixed in 3% glutaraldehyde dissolved in 0.1 m cacodylate buffer (pH, 7.4), containing 5% sucrose for 1 h at RT. After washing the cells several times in cacodylate buffer (pH, 7.4), samples were postfixed in 2% osmium tetroxide dissolved in 0.1 m cacodylate buffer (pH, 7.4) for 1 h at RT. Following several washes in cacodylate buffer (pH, 7.4), cells were dehydrated and embedded into Durcupan ACM resin. Ultrathin sections were collected on Formvar‐coated single‐slot grids and counterstained with uranyl acetate and lead citrate. Sections were observed with a transmission electron microscope (JEOL 1010, JEOL Ltd, Japan) and photographed at a magnification of 5000–30 000× with an Olympus Veleta CCD camera (Olympus Corp. Tokyo, Japan). Cell and mitochondria contours were manually segmented. Only mitochondria with visible and intact internal membranes were considered for consecutive analyses. Amira 3D (version 2022.1; Thermo Fischer Scientific) image analysis software was used to analyze the numerical parameters of the segmented structures as follows: visible area (μm^2^) of the cell in a given image, the total (summed) and average area (μm^2^) and inside length (μm) of mitochondria within the visible part of the cell. The length (inside length; μm) and area (μm^2^) were determined for 5–112 individual mitochondria per cell in the mCherry control and 11–56 individual mitochondria per cell in the mCherry‐Drp1 WT and 2–45 individual mitochondria per cell in the mCherry‐Drp1 S39A cell line.

### Seahorse XF analysis

3.5 × 10^4^ cells per well of stably overexpressing mCherry control, mCherry‐Drp1 WT, and mCherry Drp1 S39A were seeded in XF96 seahorse plates (Seahorse Bioscience) with appropriate background correction wells. One hour before the assay, the medium was changed to XF DMEM (Seahorse Bioscience, Agilent, Santa Clara, CA, USA). The sensors with the calibrant solution were incubated overnight at 37 °C without CO_2_. Measurements were performed using the Seahorse XF96 Analyzer. For XF Cell Mito Stress analysis, the media was replaced with the 180 μL of XF DMEM, pH 7. 4 (cat. 03575‐100, Agilent Technologies) supplemented with 25 mm glucose, 2 mm l‐glutamine, and 1% penicillin/streptomycin and the plate was calibrated for 1 h at 37 °C in a non‐CO_2_ incubator. After a 20‐min equilibration time, OCR was measured every 6 min (1 min mixing, 5 min measurement) for five cycles. Mitochondrial inhibitors were applied at the following final concentrations: 1.5 μm oligomycin (Oligo), 1 μm FCCP (F), 1 μm antimycin‐A (Anti), and 1 μm rotenone (Rot). The OCR values were normalized to the total protein concentration of each well using a quick Bradford protein assay (Bio‐Rad Laboratories). Data were analyzed using a 2.3 agilent seahorse desktop Software.

### Sulphorhodamine B (SRB) assay

Cell viability was measured using a sulphorhodamine B (SRB) assay. The assay measures cellular protein content, as described by Vichai and Kirtikara [61]. Cell viability was calculated as follows: % cell viability = Absorbance sample/Absorbance negative control or untreated sample × 100.

### *In silico* molecular modeling

Protein information was obtained from UniProt (O00429; DNM1L_HUMAN), while post‐translational modifications (PTM) were from the PhosphoSitePlus database [62]. Ligand‐bound structures of human Drp1 protein (PDBID: 3W6N) [63] and (PDBID: 4H1V) [22] and the structure of the oligomeric protein (PDBID: 4BEJ) [64] were downloaded from RCSB Protein Data Bank [65].

We used the netphos‐3.1 online tool to predict phosphorylation sites based on protein sequence. The prediction scores range from 0 to 1. The higher the score, the higher the phosphorylation probability; the sites with < 0.5 scores are not considered phosphorylation sites [66].

### Statistical analysis

Data are presented as means and standard deviations (SDs) of *n* ≥ 3 experiments. The normality of the population was determined using the Shapiro–Wilk test. One‐way ANOVA with multiple comparisons followed by Tukey's *post hoc* tests or Kruskal–Wallis test followed by Dunn's multiple comparison *post hoc* test, and unpaired *t*‐test or Mann–Whitney test were used for analyses. Two‐way ANOVA with Tukey's *post hoc* test was used to analyze the Seahorse experiments and mitochondrial classes. Statistical analyses were conducted using graphpad prism v10.2.2 (La Jolla, CA, USA). A *P*‐value of < 0.05 was considered significant.

## Results and Discussion

### Mutation of Drp1 at Ser39 leads to a defect in mitochondrial fission, promoting the formation of fused mitochondria

**Fig. 1:**
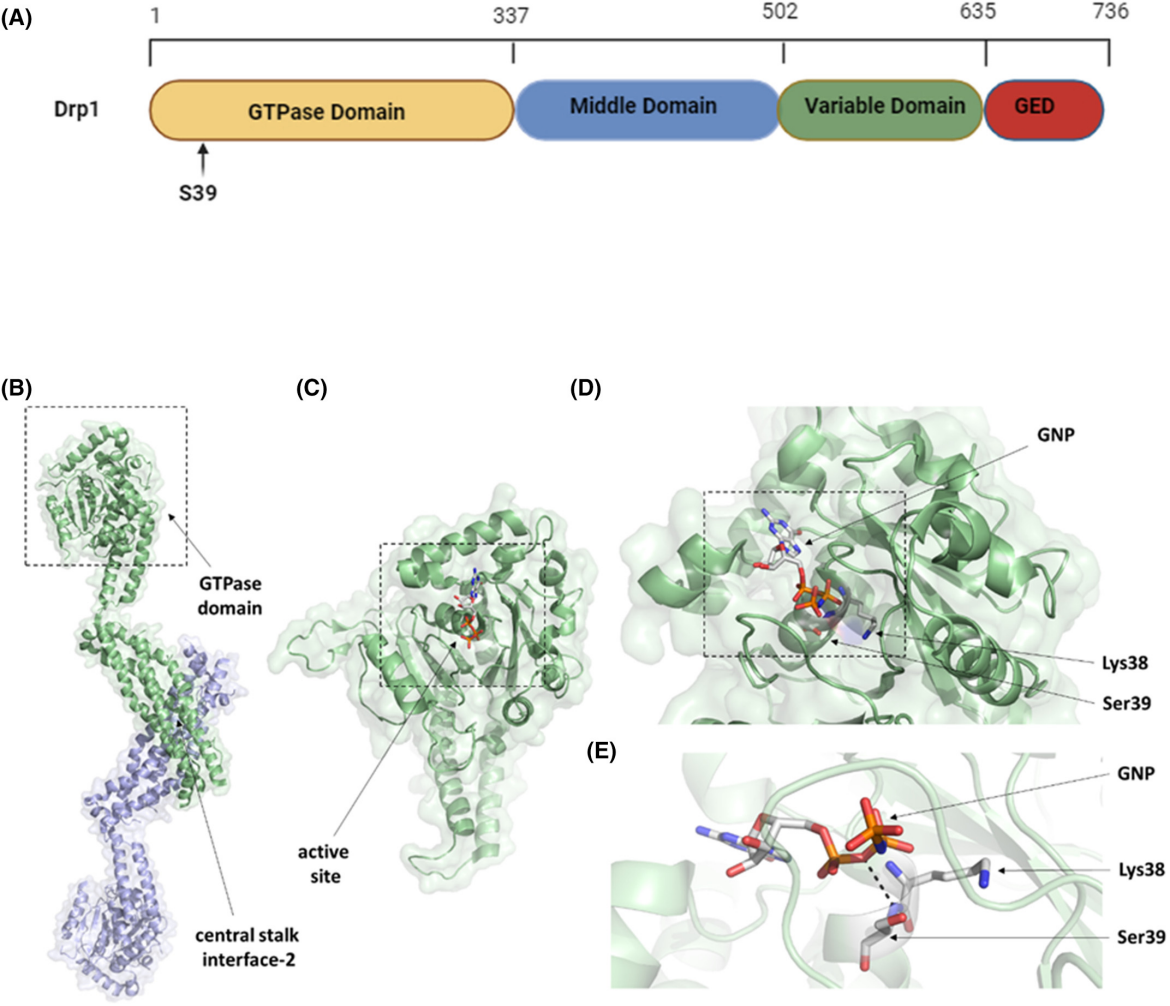
Structure of the human Drp1 protein. (A) Schematic representation of Drp1. (B) The structure of the Drp1 dimer based on its crystal structure (PDB ID: 4BEJ) [64]. The monomers are labeled with light green and blue colors. The box indicates the GTPase domain, and the dimer interface (stalk interface‐2) is also shown. (C) Enlarged view of the GTPase domain of Drp1 binding 5′‐guanylyl‐imidodiphosphate (GMP‐PNP, referred to as GNP) as a ligand (PDBID: 4H1V) [22]. The box indicates the active site enlarged in Figure part (D). (D) The GNP ligand‐bound active site. Lys38 and Ser39 residues located at the active site are shown. (E) Enlarged view of GNP ligand binding to the active site. The dotted line represents the H‐bond between the phosphate and a backbone atom of Ser39. The Lys38 residue is also represented.

Human fibroblast 5756‐Ti cells overexpressing a dominant negative mutant of Drp1 (DLP1S39N) showed a few giant reticular peroxisomes due to reduced affinity for GTP, as reported previously [26]. Functional mapping by X‐ray structural analysis of the human Drp1 confirmed that the Ser39 amino acid is necessary for the backbone interaction of Drp1 with the α‐phosphate of GTP. Ser39 is required for GTP binding (Fig. 1A), and mutation of Ser39 to alanine leads to a complete loss of GTPase activity. The Ser39 residue is essential for the protein's GTPase activity as it is located in the phosphate‐binding loop (also called P‐loop). The sequence motif (GSQSSGKSS) encompassing Ser39 residue (underlined) is highly conserved. Ser39 is involved in binding the α‐phosphate (via backbone‐mediated hydrogen bond interaction). Still, it does not interact with the β‐phosphate, unlike the Ser residues in the corresponding position in dynamin‐1 [22].

Based on data in the PhosphoSitePlus database, the S39 residue is not a PTM site and does not undergo phosphorylation. Nevertheless, the Ser40 and Ser44 residues near Ser39 are known as PTM sites. These residues can be phosphorylated by the glycogen synthase kinase (GSK) 3β and induce mitochondrial fragmentation [67]. The Ser39 residue is not exposed to the protein surface but is buried; consequently, it is hardly accessible for phosphorylation (Fig. 1B–E).

**Fig. 2:**
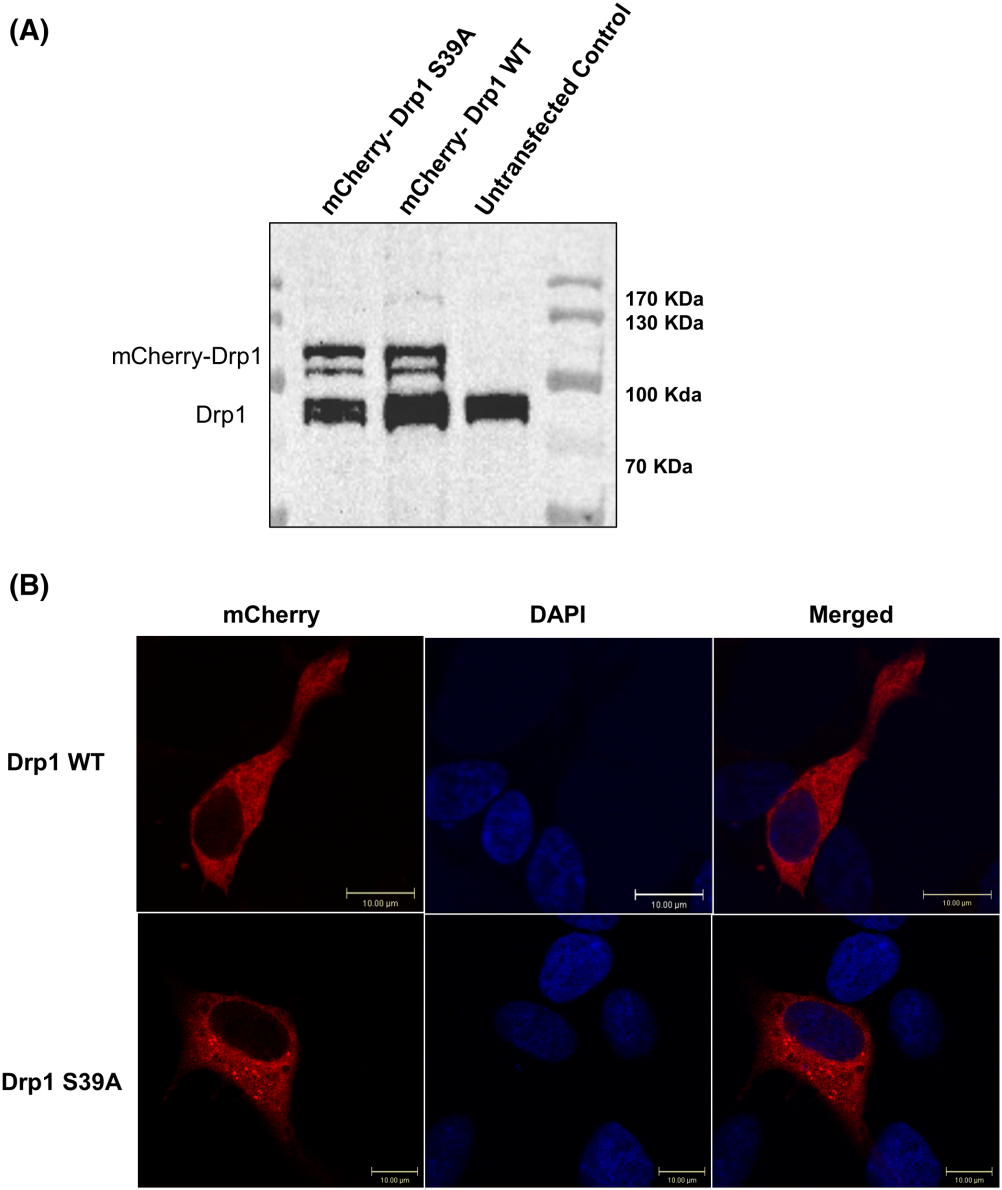
Cellular localization of the overexpressed Drp1 S39A mutant. (A) Western blot analysis confirms the overexpression of mCherry‐Drp1 WT and mCherry‐Drp1 S39A proteins in SH‐SY5Y neuroblastoma cells. An anti‐DNM1L antibody detected the endogenous Drp1, the overexpressed Drp1 WT, and Drp1 S39A mutant. (B) Representative confocal microscopy images of mCherry‐Drp1 WT and mCherry‐Drp1 S39A mutant in SH‐SY5Y neuroblastoma cells are shown. Cells were transfected with plasmids of the mCherry‐Drp1 WT and mCherry‐Drp1 S39A mutant for 72 h. The nuclei were labeled with DAPI, and the mCherry fluorescent protein is N‐terminally fused to Drp1 and Drp1 S39A, allowing direct monitoring and detection.

Keeping in mind that according to the Wenger study, the S39 mutation leads to the complete loss of the GTPase activity of Drp1 and that this activity is required to initiate mitochondrial fragmentation, we generated a mutant by Quick Change mutagenesis to substitute Ser39 with alanine (S39A) to study mitochondria. For the backbone plasmid, we used the mCherry‐Drp1 from Gia Voeltz (Addgene plasmid # 49152). Western blot analysis confirmed the overexpression of mCherry‐Drp1 S39A and mCherry‐Drp1 WT (wild‐type) in the human neuroblastoma SH‐SY5Y cell line (Fig. 2A). To check the intracellular localization of the mCherry‐Drp1 S39A mutant, we transiently transfected SH‐SY5Y cells and performed confocal microscopy analysis on fixed cells. Microscopic imaging confirmed that the Drp1 S39A mutant distribution appears with punctae (Fig. 2B).

**Fig. 3:**
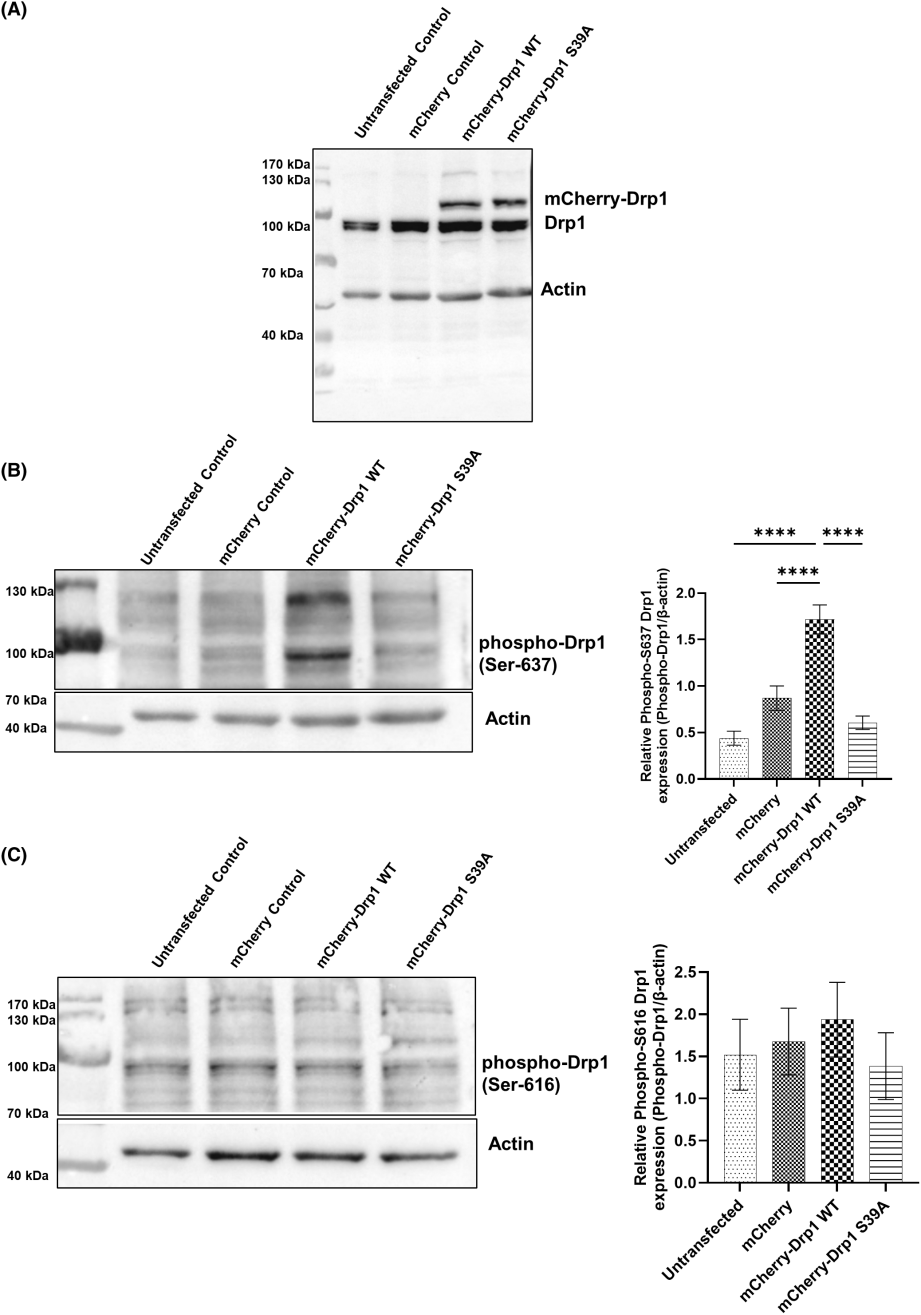
S39A Mutation of Drp1 does not impact the phosphorylation status of Ser‐616 and Ser‐637 of Drp1. RIPA buffer was used to lyse cells stably overexpressing the mCherry, mCherry‐Drp1 WT, and mCherry‐Drp1 S39A. An equal amount of protein was separated on SDS/PAGE and transferred to a nitrocellulose membrane. Unphosphorylated (A) and phosphorylated Drp1 at Ser‐637 (B) and Ser‐616 (C) were examined using anti‐Drp1 and phospho‐specific antibodies. Statistical analyses of the relative phospho proteins expressions are shown. Images were taken using a chemidoc imager, and the pixel intensity was quantified and normalized to the internal control, β‐Actin, using image lab software. Data are presented as means ± SD (*n* = 3). Groups were compared using One‐way ANOVA with multiple comparisons. Only significant differences are shown **** indicates *P* < 0.0001.

Next, we generated SH‐SY5Y neuroblastoma cell lines stably overexpressing the mCherry fusion protein referred to as mCherry control, the mCherry‐Drp1 WT, and mCherry‐Drp1 S39A. We assessed the phosphorylation status of Drp1 at Ser‐616 and Ser‐637 to see whether the loss of function mutant affects the phosphorylation of these two sites. Both the endogenous Drp1 and the mCherry fused proteins are expressed in the mCherry‐Drp1 WT and mCherry‐Drp1 S39A cell lines (Fig. 3A). The phosphorylation status of Drp1 at Ser‐637 and Ser‐616 was not changed by overexpressing the loss of function S39A mutant (Fig. 3B,C) compared with controls. Interestingly, though, overexpression of Drp1 WT increased the phosphorylation of Drp1 at Ser‐637 but not at Ser‐616 compared to the control and the mCherry‐Drp1 S39A cell lines (Fig. 3B,C).

According to the RCSB Protein Data Bank protein structure, the S39A mutation is unlikely to directly affect the Ser‐616 and Ser‐637 phosphorylation in the monomer structure. It is doubtful that the mutation at position 39 would affect the structure enough to alter phosphorylation at the distal domain (Ser‐616 and Ser‐637) and vice versa. However, there may be an indirect effect at the oligomer level since the interaction between two (or more) proteins may be more likely to be affected by the mutation. Unfortunately, we have not found data on how these amino acids are related to the Drp1 oligomer. There is good agreement between the structure‐based hypothesized effect of the S39A mutation and the western blot results because the mutation does not affect the phosphorylation of the other two side chains. However, the structural analysis does not answer why the Drp1 WT overexpression affects the phosphorylation of Ser‐637. The explanation must be sought elsewhere. We speculate that the Drp1 WT overexpression must trigger an internal control process since Drp1 would only be produced in higher amounts as a cell response mainly associated with elevated stress levels and apoptosis. The phosphorylation of Ser‐637, which reduces the catalytic activity of Drp1, may be an effort to compensate for the increased Drp1 level.

### TEM confirms elongated mitochondrial ultrastructure in cells stably expressing the Drp1 S39A mutant

**Fig. 4:**
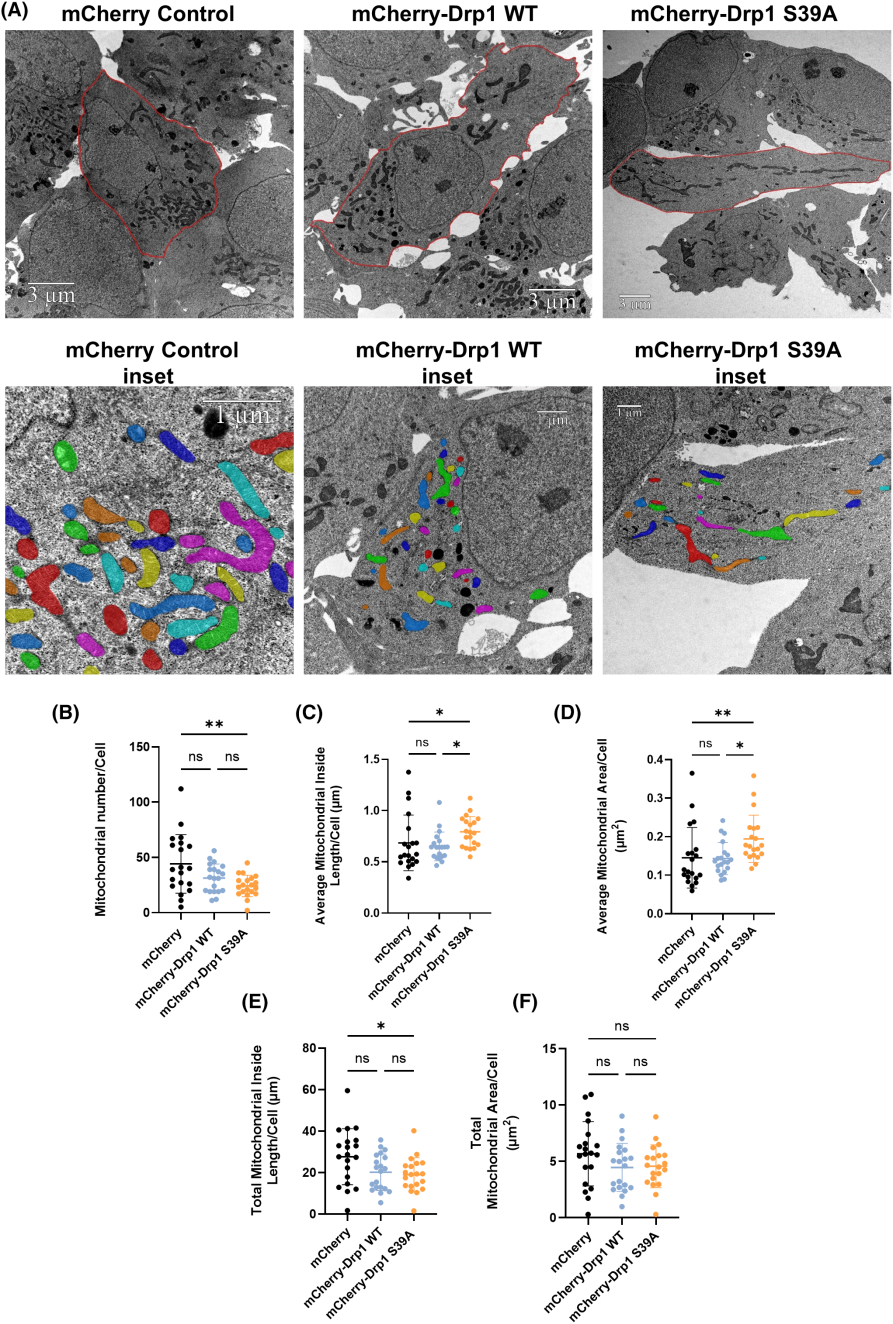
Ultrastructure of mitochondria by TEM reveals elongated mitochondrial morphology of cells stably overexpressing the Drp1 S39A mutant. (A) Cells overexpressing the mCherry control, mCherry‐Drp1 WT, and mCherry‐Drp1 S39A mutant were subject to transmission electron microscopy. Cell and mitochondria contours were manually segmented. Only mitochondria with visible and intact internal membranes were considered for consecutive analyses (insets). Mitochondria were displayed using a default shared colormaps option of Amira 3D with 8 distinct colors. Colors are independent of values; their purpose is to show the mitochondria used for further analyses. amira 3D (version 2022.1; Thermo Fischer Scientific) image analysis software was used to analyze the numerical parameters of the segmented structures. (B–F) Quantitative analyses of mitochondrial number (B), average mitochondrial inside length (μm) (C), average mitochondrial area (μm^2^) (D), total mitochondrial inside length (μm) (E), and total mitochondrial area (μm^2^) (F). One‐way ANOVA with multiple comparisons followed by Tukey *post hoc* tests or Kruskal–Wallis test followed by Dunn's multiple comparison *post hoc* test were used. Only *P* values *P* < 0.05 are considered statistically significant.

Mitochondrial homeostasis depends on fusion and fission, the division of the mitochondrion. A key regulator of fission is Drp1. Mitochondrial fission determines the mitochondria's quality, function, and morphology [7]. To test the mitochondrial morphology of cells overexpressing the Drp1 S39A, we performed transmission electron microscopy (TEM). Control cells show a mixed population of perinuclear mitochondrial ultrastructure, confirming that the overexpression of mCherry alone does not affect mitochondrial morphology and mitochondria undergo a continuous and balanced fusion–fission process (Fig. 4A). Round‐shaped and smaller‐sized mitochondrial populations with perinuclear localization were more prevalent in cells overexpressing the mCherry‐Drp1 WT (Fig. 4A). TEM imaging also demonstrates that the stable expression of mCherry‐Drp1 S39A results in a large population of elongated and giant mitochondria throughout the cell (Fig. 4A), confirming that the serine 39 amino acid residue is required to initiate GTP hydrolysis to promote mitochondrial fission. Next, we measured the number of mitochondria, the mitochondrial inside length, and the mitochondrial area using the amira 3D analysis software [40]. The number of mitochondria/cell was significantly lower in cells expressing the mCherry‐S39A mutant than in the control (Fig. 4B). The average mitochondrial inside length/cell (Fig. 4C) and the average mitochondrial area/cell (Fig. 4D) were significantly higher in cells expressing the mCherry‐Drp1 S39A mutant than in both control and mCherry‐Drp1 WT. In contrast, the average mitochondrial inside length and area of the mCherry‐Drp1 WT were not significantly different from the control (Fig. 4C,D).

On the other hand, the total mitochondrial inside length was significantly lower in the mCherry‐Drp1 S39A cell line than in the control (Fig. 4E). The total mitochondrial area did not show a significant difference (Fig. 4F). These findings suggest that while the number of mitochondria decreases, the average inside mitochondrial length increases due to fusion in cells expressing the mCherry‐Drp1 mutant. Still, the overall mitochondrial area does not change. Thus, due to a lack of fission, the Ser39 mutation significantly affects mitochondrial morphology.

### Computational analysis of mitochondrial subtype classification reveals hyperfused and round, compact tubular mitochondrial morphology in cells stably expressing the Drp1 S39A mutant

**Fig. 5:**
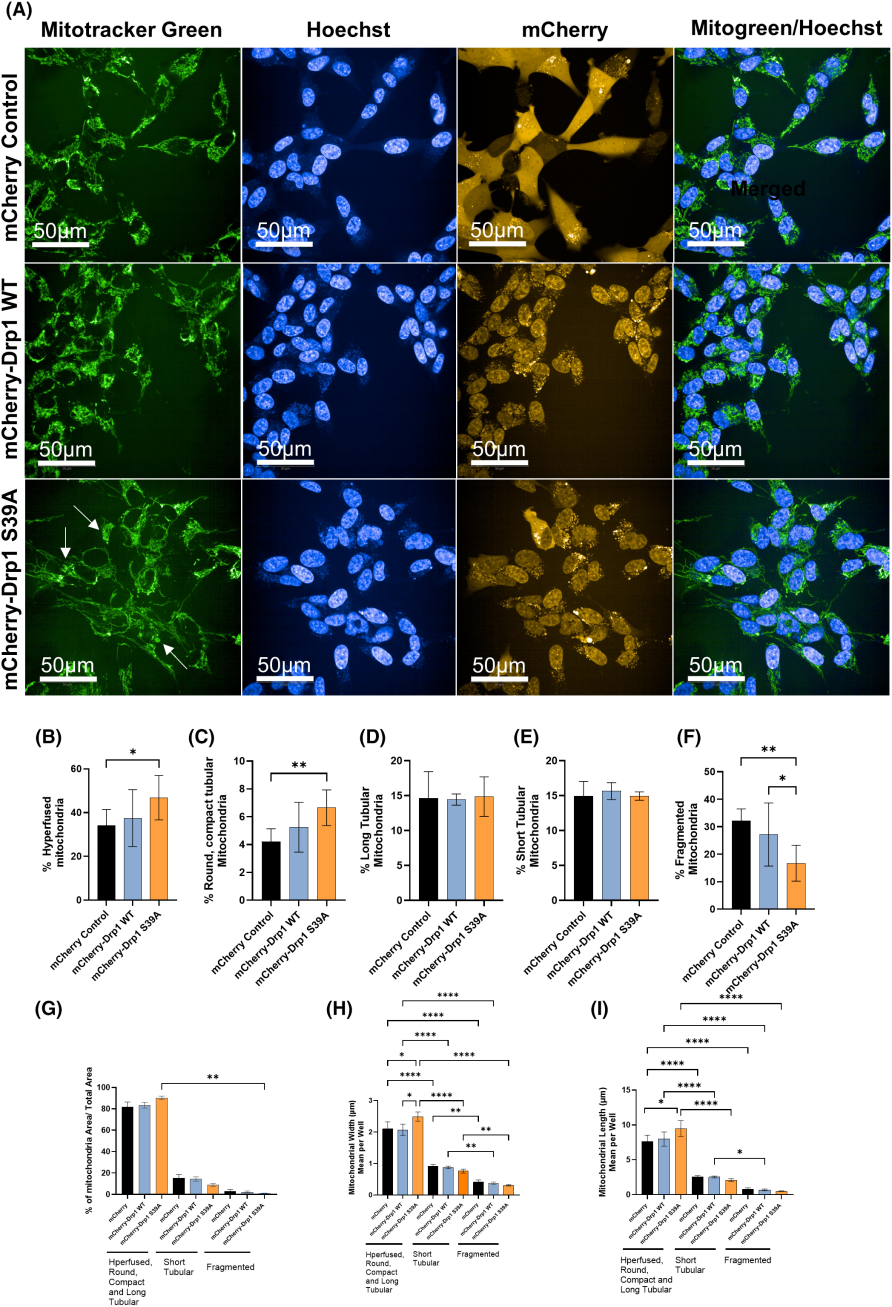
Classification of mitochondria using a high‐content‐analysis (HCA) system confirms significantly higher populations of hyperfused and round/compact tubular mitochondria in cells overexpressing the mCherry‐Drp1 S39A mutant. (A) Representative confocal images for mitochondria in live cells stained with MitoTracker Green (mitochondria labeling), Hoechst (nuclei staining), and mCherry. Cell lines stably express the mCherry control, mCherry‐Drp1 WT, and the mCherry‐Drp1 S39A (scale bar = 50 μm). Automated confocal microscopy was performed on an Opera Phenix high‐content screening system (Perkin Elmer). Image‐acquisition settings were 63× water objective (NA = 1.15), appropriate lasers, and filters for Hoechst, mCherry, and Mitotracker Green in sequential mode to exclude spectra overlap (B–F) Statistical analysis was performed by One‐Way ANOVA using multiple comparisons test, where * indicates *P* < 0.05, ** indicates *P* < 0.01. Quantitative analyses of mitochondrial classes are shown as the percent composition of each class versus all classes. (G–I) Quantitative analyses of mitochondrial properties such as total mitochondrial area (μm^2^) (G), width (μm) (H), and length (μm) (I) of each mitochondrial class per well. Data are presented as the mean ± SD of three biological replicates. Statistical analysis was performed by Kruskal–Wallis test with multiple comparisons (G) or One‐Way ANOVA using multiple comparisons test (H and I) where * indicates *P* < 0.05, ** indicates *P* < 0.01, and **** indicates *P* < 0.0001. Only significant variances are shown.

Cells stably expressing the mCherry control, mCherry‐Drp1 WT, and mCherry‐Drp1 S39A were stained with Mitotracker Green and Hoescht for live cell imaging (Fig. 5A). mCherry control and mCherry‐Drp1 WT cells showed an interconnected mitochondrial network localized mainly in the perinuclear region, similar to what we detected by TEM and to the results published by Smirnova et al. [68] (Fig. 5A). The overexpression of Drp1 WT caused shorter tubular rearrangement than in control and cells overexpressing the mCherry‐Drp1 S39A showed a mitochondrial network that collapsed into large perinuclear blebby aggregates with long, retained tubules (Fig. 5A labeled with arrows). Note that the mCherry‐Drp1 WT and the mCherry‐Drp1 S39A proteins show mainly mitochondrial localization with punctae.

Next, we classified and measured mitochondrial objects by morphology to separately evaluate the degree of morphological differences between the mCherry, mCherry‐Drp1 WT, and mCherry‐Drp1 S39A cell lines (Fig. S1A,B and Fig. 5B–I). The data also describe mean values for mitochondrial objects' morphological parameters within an individual cell. Previously, our group established an algorithm using an Opera Phenix High Content Analysis machine based on morphology and intensity properties for classification [40, 60]. Five mitochondrial types were defined: hyperfuse, round/compact tubular, long tubular, short tubular, and fragmented. In the automation of the population sorting, approximately 1500 mitochondrial objects were manually selected and classified into the five mitochondrial classes/types (Fig. S1A,B). The different proportions of mitochondrial classes in each cell line indicate the effects of the overexpression of the mCherry‐Drp1 WT or the mCherry‐Drp1 S39A proteins. We analyzed 269 000 mCherry control, 198 960 mCherry‐Drp1 WT, and 116 300 mCherryDrp1 S39A cells in three independent experiments. The percentage of each mitochondrial class was calculated by the following formula: (mitochondrial class area (μm^2^)/total mitochondria area (μm^2^) × 100). The quantitative analysis determined that mCherry‐Drp1 S39A cells have a significantly higher percentage of hyperfused and round/compact tubular mitochondria than the mCherry control (Fig. 5B,C). Long and short tubular morphology was similar in all three cell lines (Fig. 5D,E), and no significant differences were detected.

In contrast, fragmented mitochondria were significantly lower in the mCherry‐Drp1 S39A mutant than in the mCherry control and mCherry‐Drp1 WT (Fig. 5F). The morphology parameters analyzed by HCS confirmed the TEM data. We combined the three classes—hyperfused, round/ compact tubular, and long tubular—representing fused mitochondrial morphology. Mitochondria areas were not significantly different among the cell lines of the same mitochondrial class (Fig. 5G). The mean mitochondrial width (Fig. 5H) and length (Fig. 5I) of classes representing fused mitochondria were significantly higher in cells expressing the mCherry‐Drp1 S39A compared with the mCherry control and mCherry‐Drp1 WT.

### Cells with the Drp1 S39A mutation show similar mitochondrial bioenergetics and viability to control

**Fig. 6:**
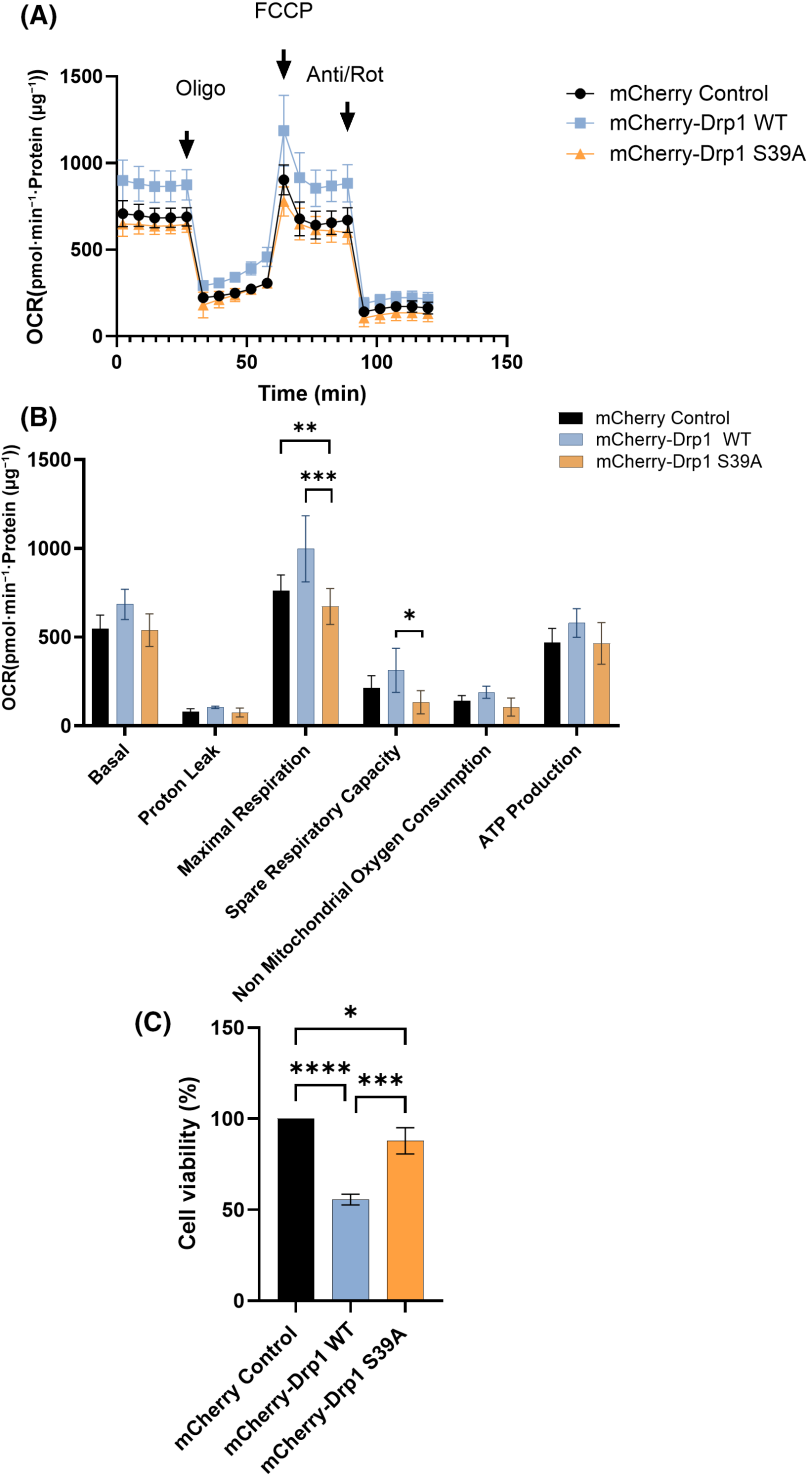
Mitochondrial bioenergetics analysis of cells stably expressing the mCherry‐Drp1 S39A mutant shows a similar profile to that of the control. (A) Oxygen consumption rate (OCR) was measured using the Seahorse XF 96 to analyze mitochondrial function. Cells were seeded at a 35 000 cell per well density in Seahorse XF 96 plates. The following day, the basal OCR was determined for 30 min after sequentially injecting mitochondrial drugs, including 1.5 μm oligomycin (Olig), 1 μm FCCP, and 1 μm of rotenone/antimycin‐A cocktail (Anti/Rot). (B) Calculated basal respiration, proton leak‐linked respiration, maximal respiration, spare respiratory capacity, non‐mitochondrial respiration, and ATP‐coupled respiration. Data were normalized to total protein (pmol·min^−1^·μg^−1^ protein). Data were analyzed using wave desktop software and presented as mean values ± SD (*n* = 3). Statistical analysis was performed with graphpad prism v10.2.2 software by Two‐Way ANOVA using multiple comparison tests (* indicates *P* < 0.05, ** indicates *P* < 0.01, and *** indicates *P* < 0.001). (C) Cell viability was assessed by an SRB assay in cells stably expressing the mCherry, mCherry‐Drp1, and mCherry‐Drp1 S39A proteins. Results are presented as the mean ± SD of three independent experiments. Statistical analysis was performed by One‐Way ANOVA using graphpad prism v.10.2.2 (* indicates *P* < 0.05, *** indicates *P* < 0.001, and **** indicates *P* < 0.0001).

The mitochondria are vital organelles with a critical role in cellular ATP production. ATP is an energy source, especially in cells and organ systems with high metabolic activity. Mitochondrial dysfunction may result from somatic or mitochondrial DNA (mtDNA) mutations, fusion and fission cycle disruption, and inherited mitochondrial dysfunction [69, 70, 71, 72]. Mitochondrial dynamics changes are linked to metabolic adaptation in healthy cells [73, 74, 75, 76, 77]. However, aberrant changes in both are closely correlated with certain diseases, including aging and neurodegenerative disorders [70, 78, 79, 80, 81, 82, 83]. We tested the mitochondrial bioenergetics profile using the Cell Mito Stress assay by Seahorse XF Analysis. In real time, we measured oxygen consumption rate (OCR) in cells stably overexpressing mCherry, mCherry‐Drp1 WT, and mCherry‐S39A recombinant proteins. Basal OCR and OCRs were measured after adding selective mitochondrial inhibitors in the following sequential order: oligomycin, carbonyl cyanide‐4‐(trifluoromethoxy)phenylhydrazone (FCCP), and a combination of rotenone and antimycin A (Fig. 6A). Oligomycin inhibits ATP synthase and prevents proton entry into mitochondria. Maximal respiration (uncoupled mitochondrial respiration) was measured by adding FCCP. FCCP reduces ATP synthesis by collapsing the proton gradient across the inner mitochondrial membrane. To distinguish between mitochondrial and non‐mitochondrial oxygen consumption, we used the complex I inhibitor rotenone and the complex III inhibitor antimycin A to inhibit mitochondrial respiration. OCR was normalized to total protein content. The results showed that overexpressing Drp1 significantly increased the OCR‐based maximal respiration compared to the control (Fig. 6B). Maximal respiration is the maximum rate measurement after FCCP injection—nonmitochondrial respiration (basal + spare capacity). The significant increase in maximal respiration was due to a significantly increased level of spare capacity. The OCR‐based calculated ATP production, however, was similar to the control. The increase in spare respiratory reserve is indicative of stress. When cells are subject to stress, energy needs increase, and ATP production increases to maintain cellular homeostasis. Our data suggest that the overexpression of Drp1 triggers a stress response where cells try to cope with increased spare reserve, showing an increasing but not significant trend of ATP production. Intriguingly, we found that overexpressing the Drp1 S39A mutant reversed the effect of Drp1 overexpression and reduced both the maximal respiration and spare capacity to the control level (Fig. 6B).

Overexpression of Drp1 significantly increases the proliferation and apoptosis rate of the 95D human lung cancer high metastatic and A549 lung adenocarcinoma cell lines, suggesting that inhibition of Drp1 expression might contribute to antitumor therapy [84]. Inhibition of Drp1 function by overexpressing the dominant‐negative K38A mutant blocked apoptotic cell death in COS‐7 and SW480 human colon adenocarcinoma cells, highlighting the importance of mitochondrial fission in apoptosis [85]. Thus, we assessed a cell viability assay and found that overexpression of Drp1 WT sensitizes cells to cell death (a significant 44% decrease in cell viability). In contrast, in the dominant‐negative mutant, viability was similar to control (12% decrease in viability). This result suggests that the dominant‐negative S39A mutation has a similar effect to the K38A mutation and that the overexpression and hyperactivation of Drp1 may initiate cell death (Fig. 6C) [46]. A study demonstrated that expression of Drp1 sensitized cells to different apoptotic insults and suppression of mitochondrial fission by down‐regulating Drp1, conversely, reduced but did not prevent Bax/Bak‐dependent apoptotic cell death in HeLa cells [86]. Recently, it was proposed that Drp1 promotes apoptosis as a noncanonical activator of Bax via N‐terminal binding. Forced dimerization of Bax and Drp1 promotes their activation and induces their translocation to the mitochondria, mitochondrial remodeling, and apoptosis without apoptotic stimuli [87]. Furthermore, Drp1 was shown to be involved in Bak activation, facilitating OMM breaks, cytochrome c release, and apoptosis independently of mitochondrial morphology perturbation [88]. Together, these data suggest that the function of Drp1 in apoptosis depends on specific insults, cell type, and death pathway.

## Conclusions

This study demonstrated that the serine 39 residue of Drp1 is likely to regulate mitochondrial morphology rearrangement, bioenergetics, and cell death. This site was functionally mapped by X‐ray structural analysis by Wenger *et al*. [22]. The group identified the Ser39 site as part of the phosphate‐binding loop in the GTPase domain of Drp1 and that the mutation of S39A resulted in the complete loss of GTPase activity and exhibited no GTP turnover. The Ser39 residue is required for backbone interaction between the α‐phosphate of GTP and Drp1. Based on an identified dominant‐negative dynamin‐specific genetic mutation S45N [89], Li and Gould [26] generated a corresponding S39N mutant with reduced affinity to bind GTP. It inhibited peroxisome division and decreased abundance in human fibroblasts. Keller *et al*. [56] reported in a clinical study about a 10‐year‐old male with sensory neuropathy that resulted in severe muscular atrophy, and that was due to a *de novo* mutation of Drp1 at serine 39 to glycine. The functional consequence and the pathogenesis of the mutation are known, but what happens at the cellular level, mainly with mitochondrial morphology, which Drp1 primarily regulates, is unknown. The functional defects caused by mutations in Drp1 are highly variable, even for mutations located within the same functional domain, highlighting the complexity of Drp1 regulation [57]. Drp1 and its GTPase activity are essential for mitochondrial division; we therefore investigated mitochondrial morphology and metabolism in an *in vitro* cellular model. We demonstrated by TEM and quantitative computational analysis that the S39A mutation of Drp1 results in a highly fused, elongated, and clustered tubular mitochondrial phenotype, with a reduced number of mitochondria but maintained area size compared to control. Mitochondrial bioenergetics were similar to the control, and cell death was inhibited in the S39A mutant compared to cells overexpressing the wild‐type Drp1 WT. We propose that the serine 39 residue of Drp1 is required to distribute mitochondria in a cell through its involvement of the GTPase activity and that the amino acid mutation leads to structural anomalies in the mitochondrial network. The study provides additional data to gain insight into the comprehensive understanding of the function of the Drp1 protein.

## Conflict of interest

The authors declare no conflict of interest.

### Peer review

The peer review history for this article is available at https://www.webofscience.com/api/gateway/wos/peer‐review/10.1002/2211‐5463.13820.

## Author contributions

MG performed cell biology experiments, bioenergetics experiments, and western blotting, performed data analysis and interpretation; BS performed molecular biology experiments, quick change mutagenesis; MA was involved in the acquisition of data, data analysis, assisted in writing the first draft of the manuscript; HA was involved in experimenting; PZ assisted in writing the first draft of the manuscript; EAJ was involved in statistical analyses; JAM was involved in modeling of Drp1 structure, prediction of phosphorylation KT, conception and design, analysis, and interpretation of data, generation of stable cell lines, primer design, quick change mutagenesis, supervision, acquiring fund, drafting, writing and editing the manuscript.

## Supporting information


**Fig. S1.** Classification of mitochondrial morphology.

## Data Availability

Data supporting reported results can be found at the Department of Medical Chemistry http://193.6.152.202:5000/. Requests to access the datasets should be directed to the corresponding author.
