# Supplementary material for: Serine 39 in the GTP‐binding domain of Drp1 is involved in shaping mitochondrial morphology
Source: FEBS Open Bio. 2024 May 17;14(7):1147–65. doi: 10.1002/2211-5463.13820 (PMC11216946; doi:10.1002/2211-5463.13820)
Supplement: Supplementary file 1 — Fig. S1. Classification of mitochondrial morphology. [file FEB4-14-1147-s001.docx]

**
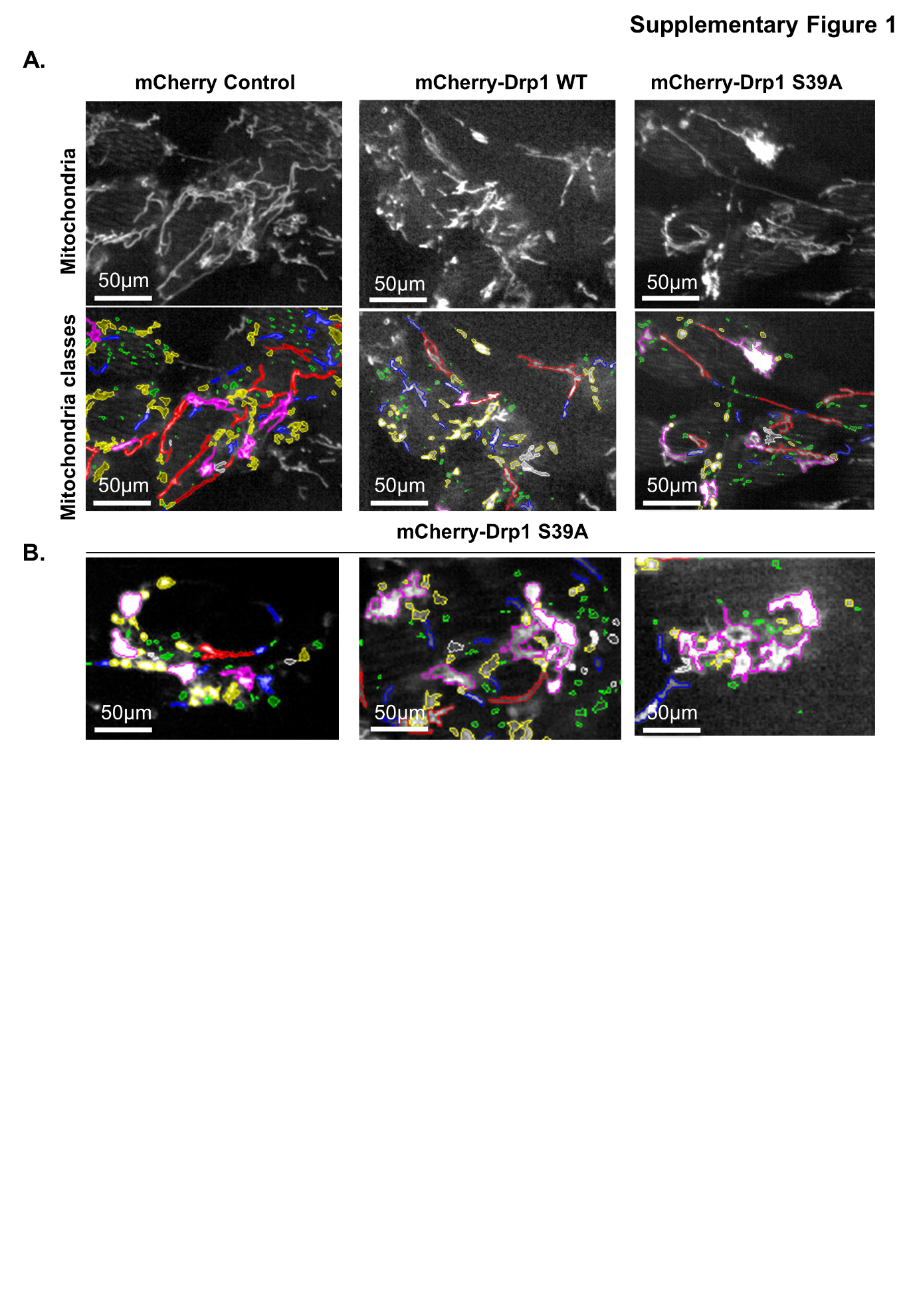
Supplementary Figure 1. Classification of mitochondrial morphology. (A)** Mitochondria species were classified using the built-in Harmony 4.9 and PhenoLogic machine-learning software. Mitochondria were classified as hyperfused-pink; round/compact tubular-yellow; long tubular-red; short tubular-blue; and fragmented-green. **(B)** Representation of mitochondrial classes in mCherry-Drp1 S39A cells.
